# Estimating the rice nitrogen nutrition index based on hyperspectral transform technology

**DOI:** 10.3389/fpls.2023.1118098

**Published:** 2023-03-22

**Authors:** Fenghua Yu, Juchi Bai, Zhongyu Jin, Honggang Zhang, Jiaxin Yang, Tongyu Xu

**Affiliations:** School of Information and Electrical Engineering,Shenyang Agricultural University, Shenyang, China

**Keywords:** UAV hyperspectral, NNI, critical nitrogen concentration, BES-ELM, rice

## Abstract

**Background and objective:**

The rapid diagnosis of rice nitrogen nutrition is of great significance to rice field management and precision fertilization. The nitrogen nutrition index (NNI) based on the standard nitrogen concentration curve is a common parameter for the quantitative diagnosis of rice nitrogen nutrition. However, the current NNI estimation methods based on hyperspectral techniques mainly focus on finding a better estimation model while ignoring the relationship between the critical nitrogen concentration curve and rice hyperspectral reflectance.

**Methods:**

This study obtained canopy spectral data using unmanned aerial vehicle (UAV) hyperspectral remote sensing and determined the rice critical nitrogen concentration curve and NNI. Taking the spectrum at critical nitrogen concentration as the standard spectrum, the original spectral reflectance and logarithmic spectral reflectance data were transformed by the difference method, and the features of the spectral data were extracted by a Autoencoder. Finally, the NNI inversion models of rice based on Extreme Learning Machine (ELM) and Bald Eagle Search-Extreme Learning Machine (BES-ELM) were constructed by taking the feature bands of four spectral extractions as input variables.

**Results:**

1) from the feature extraction results of the self-encoder, simple logarithmic or difference transformation had little effect on NNI estimation, and logarithmic difference transformation effectively improved the NNI estimation results; 2) the estimation model based on the logarithmic difference spectrum and BES-ELM had the highest estimation accuracy, and the coefficient of determination (R2) values of the training set and verification set were 0.839 and 0.837, and the root mean square error (RMSE) values were 0.075 and 0.073, respectively; 3) according to the NNI, the samples were divided into a nitrogen-rich group (NNI ≥ 1) and nitrogen-deficient group (NNI < 1).

**Conclusion:**

The logarithmic difference transformation of the spectrum can effectively improve the estimation accuracy of the NNI estimation model, providing a new approach for improving NNI estimation methods based on hyperspectral technology.

## 1 Introduction

Rice is one of the most important food crops in Asia. Nitrogen is an important element affecting the growth, development, and yield of rice. A change in nitrogen content will affect the photosynthesis, protein production, and carbon and nitrogen metabolism of crops (Jiaying et al., 2022). Nitrogen can inhibit the growth of the aboveground parts and roots of crops, limit the formation and development of the reproductive organs, and significantly affect the yield and quality of crops (Hu et al., 2023). Therefore, diagnosing the nitrogen demand in rice fields quickly, accurately, and in a large area is an essential means to realize accurate management and fertilization in rice fields and ensure adequate yield (Lemaire et al., 2008).

Currently, research has mainly focused on obtaining nitrogen content data from rice. However, it remains difficult to provide quantitative guidance on nitrogen abundance and deficiency simply based on the nitrogen content of rice. The critical nitrogen concentration curve theory is the leading standard for rice nitrogen nutrition (Song et al., 2020). Greenwood et al. first proposed the essential concept of nitrogen concentration; that is, the minimum nitrogen concentration required by crops to reach maximum biomass (Greenwood et al., 1991). Then Lemaire constructed the nitrogen nutrition index (NNI) (Lemaire et al., 1996) by calculating the ratio of the actual nitrogen content of plants to the corresponding critical nitrogen concentration to make better use of the necessary nitrogen concentration curve in nitrogen nutrition diagnosis (Lemaire et al., 1996). As an essential index of crop nitrogen nutrition diagnosis, the NNI can quantitatively describe the nitrogen nutrition abundance and deficiency of crops, provide an adequate crop nitrogen nutrition diagnosis, and provide quantitative guidance for subsequent fertilization decision-making. Bo used the critical nitrogen concentration curve of indica rice that had been planted and cultivated using mechanized agricultural practice to determine its NNI and cumulative nitrogen deficit, and then evaluated its nitrogen nutrition status, which was used as a guide for fertilization (Bo et al., 2021).

However, the traditional calculation of the NNI requires nitrogen content and biomass data obtained through field sampling, which is high in cost, has a long measurement period, and delayed results (Liu et al., 2019), and cannot be applied to actual crop production. By using a hyperspectral camera on an Unmanned Aerial Vehicle (UAV) platform to obtain ground spectral information, rice spectral data from a large area can be obtained in a short time (Hiroyuki et al., 2010; Yuanyuan et al., 2021). In recent years, with the development of airborne hyperspectral technology, the use of UAV hyperspectral remote sensing technology to obtain physical and chemical information on rice has been an important development in the field of precision agriculture (Chanseok et al., 2011). Hyperspectral remote sensing technology can obtain the growth information of crops by analyzing their spectral data and has the advantages of being fast, accurate, and with only a slight loss of information (Yongguang et al., 2021). One study used eight vegetation indexes from a rice canopy spectrum in a cold region as input and compared the inversion effects of different machine-learning algorithms, and found that the support vector regression based on the binary particle swarm optimization algorithm (BPSO-SVR) algorithm had the best inversion effect, with coefficient of determination (R^2^) values of 0.913–0.949 and root mean square error (RMSE) values of 0.055–0.127 (Kezhu et al., 2018). Shi et al. estimated the dry weight, leaf area index, and nitrogen accumulation of a rice canopy by obtaining RGB images, and found that the regression model based on the random forest algorithm had the best accuracy and generalization ability (Peihua et al., 2021).

Therefore, the combination of UAV hyperspectral remote sensing technology and critical nitrogen concentration curve theory, and the method of retrieving the NNI from hyperspectral remote sensing data to diagnose rice nitrogen nutrition, is currently an important research hotspot (Ata-Ul-Karim et al., 2017; Yu et al., 2021). Haynie et al. obtained rice canopy spectral data through uncrewed aerial vehicle remote sensing, constructed a variety of vegetation indexes, and combined them with a machine learning algorithm to model the inversion of rice aboveground biomass, nitrogen uptake, and NNI. The results showed that the random forest algorithm significantly improved the inversion accuracy of the NNI (Hainie et al., 2020). Qiu et al. extracted RGB spectral information from rice and constructed a vegetation index. Comparison of the accuracy of the retrieved NNIs of rice by various machine learning methods indicated that the random forest algorithm performed best in each growth period (Zhengchao et al., 2021).

The current method of NNI estimation by remote sensing is similar to that of N content estimation, mainly by combining spectral feature extraction and machine learning technology to improve the accuracy of NNI estimation, while the relationship between NNI and N content of rice is not purely positive, and the NNI and N content of different fertility periods cannot correspond to each other, and even the same sampling period may fluctuate according to the development of individual rice, therefore, the estimation of NNI by canopy spectra requires targeted processing of rice canopy spectra of different fertility periods to highlight the characteristics of rice canopy spectra under different NNI.Therefore, the current study used the hyperspectral data of rice fields obtained by uncrewed aerial vehicles and NNI as the research objects and took the canopy spectrum of NNI ≈ 1 in different periods as the standard spectrum. The difference between the original hyperspectral data and logarithmic spectral data of rice was then amplified by difference transformation, and a self-encoder algorithm was used to process the initial spectral curve and transform the spectra, extract the transform spectral features, and establish rice NNI estimation models based on Extreme Learning Machine (ELM) and Bald Eagle Search-Extreme Learning Machine (BES-ELM). Finally, the estimation accuracy of the models was compared, and the best estimation method of rice NNI based on hyperspectral data was determined to provide a new research approach for rapidly obtaining rice NNI.

## 2 Materials and methods

### 2.1 Experimental design

The experiment was conducted from June to September, 2021 at the Precision Agriculture Aviation Research Base of Shenyang Agricultural University (40 58’ north latitude and 122 43’ east longitude) in Gengzhuang Town, Haicheng City, Anshan City, Liaoning Province. To avoid remote sensing data collection error caused by rainy and cloudy weather, data collection proceeded only on fine days. The collection was postponed if the cloud cover exceeded 20% or if the weather was unfavorable for remote sensing data collection. The rice variety used was ‘Beijing 1705’. The experimental field was divided into two large areas. In Experiment 1, five nitrogen gradients were set up, and the nitrogen application rates were N0 = 0 kg.hm^-2^, N1 = 75 kg.hm^-2^, N2 = 150 kg.hm^-2^, N3 = 225 kg.hm^-2^, and N4 = 300 kg.hm^-2^. In Experiment 2, five nitrogen gradients were designed, and the nitrogen application rates were N0 = 0 kg.hm^-2^, N1 = 50 kg.hm^-2^, N2 = 100 kg.hm^-2^, N3 = 150 kg.hm^-2^, and N4 = 200 kg.hm^-2^. The area of each plot in Experiment 1 was 5*8=40m^2^, and that in Experiment 2 was 660 m^2^. Except for the gradient of nitrogen fertilizer, the field management of the two experimental areas was consistent. The application rate of phosphorus and potassium fertilizer was the local standard, in which the standard application rate of phosphorus fertilizer was 144 kg.hm^-2^, the standard application rate of potassium fertilizer was 192 kg.hm^-2^, and the ratio of base to topdressing was 1: 1. The other field management practices were the same as for conventional high-yield management. Field sampling was conducted from the tillering stage to the heading stage, and the sampling interval was nine days. Each sampling in each experimental plot selected in each experimental plot randomly selected a 1 m × 1 m plot, and a plastic frame was used for subsequent ROI identification. Three representative holes were used to obtain nitrogen concentration and aboveground Biomass(AGB), and the average value was used to determine the nitrogen concentration and AGB in the plot. In Experiment 1, 120 groups of cell data were collected, and in Experiment 2, 88 groups of cell data were collected, totaling 208 groups of samples.

### 2.2 Data acquisition

#### 2.2.1 Acquisition of spectral parameters in rice

**Figure 1:**
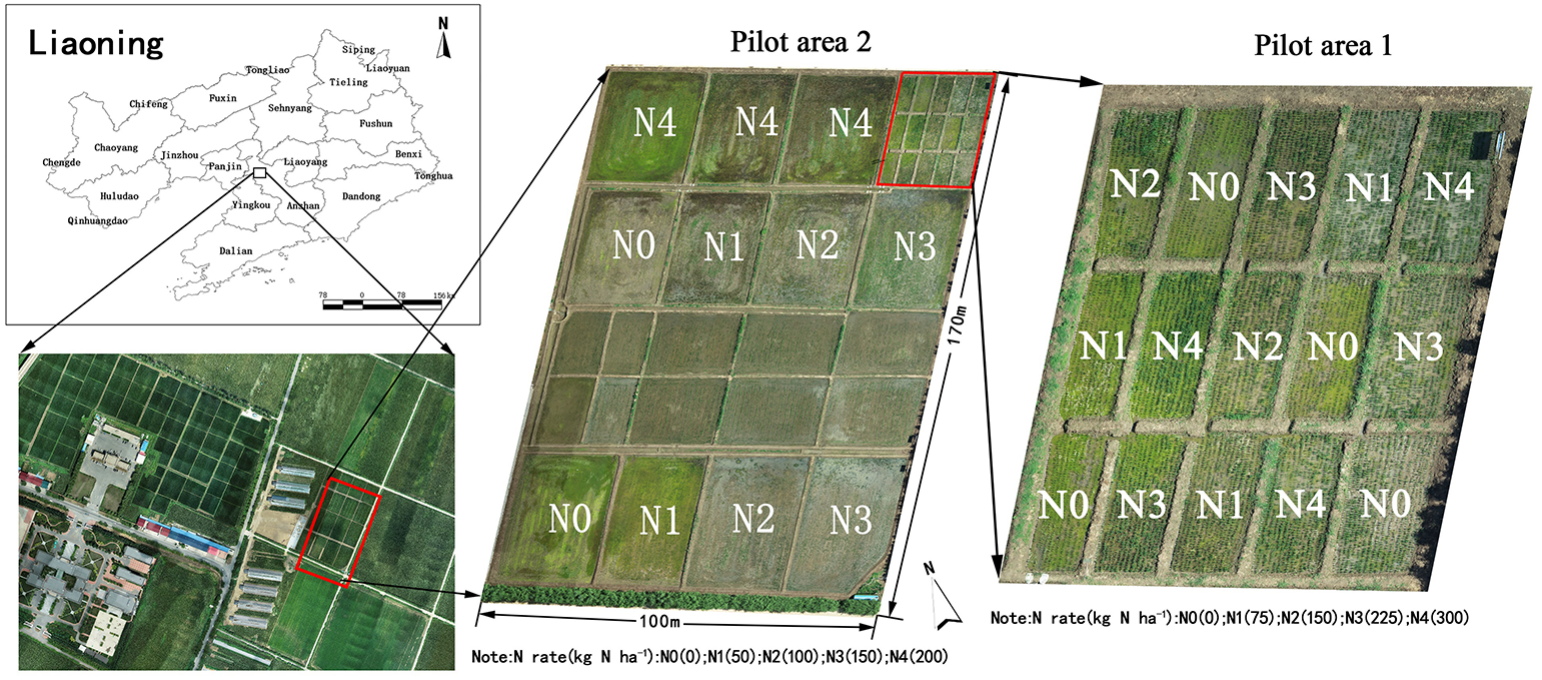
Plot distribution of experimental field 2.

The UAV hyperspectral platform adopted the M600 PRO six-rotor UAV of Shenzhen DJI Innovation Company, and the hyperspectral imager used the GaiaSky-mini built-in push-sweep airborne hyperspectral imaging system of Sichuan Shuangli Hepu Company. The hyperspectral band range was 400–1,000 nm, the resolution was 3 nm, and the number of influential bands was 253. The hyperspectral imaging system is shown in **Figure 1**. The data collection time of the UAV hyperspectral remote sensing platform was from 11:00 am to 12:00 am for each test, and the time when the sunlight intensity is relatively stable was selected. The flying height of the UAV was 100 m. Using the ENVI5.6 + IDL tool software, the hyperspectral data of the obtained hyperspectral remote sensing images were extracted. The spectral angle mapping method was used to remove the influence of interference with the spectrum of ground objects, and average spectra were calculated for the region of interest of each cell, and then the average spectra were resampled with MATLAB software to resample the spectral resolution to 1 nm. Finally, the resampled spectra were denoised by a Gaussian filter, and the results were used as hyperspectral information for each experimental cell.

#### 2.2.2 Acquisition of the agronomic parameters of rice

When measuring the nitrogen concentration of the samples and the AGB, the rice in each plot was sampled destructively at first. The pieces were brought to the laboratory and placed into an oven to deactivate the enzymes at 105°C for 30 min and then dried to constant weight at 80°C. The AGB of the dried sample was then measured and converted into theAGB part according to the planting density. Finally, the dried samples were ground, and the plant nitrogen concentration was measured using the Kjeldahl nitrogen determination method (Nelson and Sommers, 1962). The calculation formula was as follows:

(1) $$N_{c}=\frac{V\times 0.05\times 0.014}{M}\times 100$$

Nc is the sample nitrogen concentration (%), V is the volume of hydrochloric acid solution (mL), and M is the sample mass.

### 2.3 Data processing

#### 2.3.1 Critical nitrogen concentration curve and NNI calculation method

Critical nitrogen concentration is the minimum nitrogen concentration needed by crops to reach maximum biomass, and so the nitrogen deficiency equation can be constructed based on the critical nitrogen concentration to determine the nitrogen deficiency of rice. According to the critical nitrogen concentration curve method proposed by Justes et al. (1994), the critical nitrogen concentration curve was constructed based on the measured nitrogen concentration and AGB. The specific methods were as follows: (1) comparing the measured nitrogen concentration and AGB under different nitrogen fertilizer gradients, the samples were divided into a nitrogen-restricted group and non-restricted group by variance analysis. This was followed by (2) linear fitting of AGB and nitrogen concentration for the nitrogen-restricted group. (3) For the group not restricted by nitrogen nutrition, the average value of the AGB in the same period represented the maximum AGB in that period. (4) The theoretical, critical nitrogen concentration point in each period was taken as the cut-off point of the position of the fitting curve in the maximum AGB. Finally, (5) the critical nitrogen concentration points of each period were fitted by a power function, and the critical nitrogen concentration curve of the crops was constructed:

(2) $$N_{c}=a*DM^{-b}$$

In the equation, Nc is the critical nitrogen concentration (%) of rice, DM is the AGB (t/hm^2^), and a and b are both curve parameters.

The N nutrition index is the ratio of the actual nitrogen concentration to the critical nitrogen concentration in rice, and the formula was as follows:

(3) $$\text{NNI}=N_{nc}/N_{c}$$

In the formula, *N_nc_* represents the actual nitrogen content (%) of the plant under different nitrogen application rates, and Nc is the critical nitrogen concentration (%) corresponding to the plant biomass.

#### 2.3.2 Hyperspectral data processing

As the aboveground nitrogen nutrition status and canopy leaf structure of crops reach the best state under the critical nitrogen concentration, these are the direct factors affecting the spectral reflectance of the rice canopy (Lemaire et al., 2008; Lemaire et al., 2019). Therefore, compared with crops under non-critical nitrogen concentration, crops under critical nitrogen concentration have specific differences in spectral level and nitrogen nutrition status. To highlight the difference between rice crops under a critical nitrogen concentration and those under a non-critical nitrogen concentration at the spectral level, the spectral reflectance corresponding to samples with 0.99<NNI<1.01 was selected as the standard spectrum in this study. If there were multiple samples, the average value of reflectance of all samples was removed, and the difference transformation was done with the original spectra of the remaining samples in the same period. Meanwhile, considering the fact that due to the large difference in the reflectance scale of the rice canopy spectra in different bands, the simple difference transformation may lead to the blurring of the spectral features and increase the difficulty of the subsequent NNI-related feature extraction, this study adopts the logarithmic transformation to preprocess the original rice spectra and perform the difference transformation at the same time, which is calculated as follows:


(4) $$R_{ln}=\ln R_{c}$$

(5) $$R_{D}=R_{c}-R_{nc}$$

(6) $$R_{lnD}=\ln R_{c}-\ln R_{nc}$$

In the equation, *R_ln_, R_D_, R_lnD_* represent the log spectral reflectance, difference spectral reflectance, and log difference spectral reflectance, respectively; *R_nc_* represents the spectral reflectance of the sample under the critical nitrogen concentration state; and Rc represents the remaining spectral reflectance of the same period.

Due to the high similarity of data in adjacent bands of the full-band spectrum and a large amount of redundant information unrelated to the variables sought, modelling based on the full-band range often has some shortcomings, such as a slow running speed, high model error, and low inversion accuracy (Zhang et al., 2019; Jingcheng et al., 2020; Shaomin et al., 2020). To reduce the number of inputs, decrease data redundancy, and improve modelling speed and accuracy, this study used Autoencoder (AE) to extract features from spectral reflectivity data. AE is a typical unsupervised learning algorithm that automatically learns the essential features of data by compressing and reconstructing a large amount of unlabeled data. AE consists of an encoder and a decoder. The encoder compresses the input data into a low-dimensional hidden space representation, and this efficient representation of the input data is called encoding. The decoder then reduces the encoded hidden-space representation to the original sample, and trains the network by comparing the difference between the original data and the reconstructed data being reduced, passing the error between the two backwards so that the representation on the low-dimensional space can characterize the original input data. Thus, through continuous iteration, the AE can learn the internal features of the spectral data and decode the features into a high-dimensional feature vector to achieve feature extraction of the spectral data. At the same time, considering that the AE as an unsupervised algorithm lacks NNI as a label-assisted algorithm operation, the feature extraction results may have some information unrelated to the nitrogen deficit, this study adopts multiple linear regression modeling of the AE feature extraction results and the corresponding nitrogen deficit data, and the best feature extraction results are selected according to the RMSE of the regression results by eliminating the feature matrices with poor feature extraction combination for subsequent modeling.

#### 2.3.3 Inversion modelling of nitrogen deficit

In this study, two algorithms, namely ELM and BES-ELM, were selected for modelling. The ELM model was mainly used to test the optimization effect of BES. ELM is a new feed-forward type of neural network proposed by Huang et al. (2006). ELM has improved upon the problems of a slow learning speed and low generalization ability of traditional back propagation (BP) neural networks. The ELM network structure has a strong network learning ability, and compared with other traditional neural networks using the gradient descent method, the network training time of the ELM network structure is significantly shortened, while the accuracy of the running results is higher. ELM training only needs to calculate the connection weights between the hidden layer and the output layer, while other weights and biases are randomly generated at the time of network establishment and are not changed. Since the ELM algorithm has the advantages of a fast learning speed, simple structure, easy implementation, and high learning accuracy, it has a faster running speed and higher learning accuracy in dealing with more complex nonlinear problems. However, since the connection weights between the input layer and the hidden layer and the thresholds of the neurons in the hidden layer are randomly generated in each run of the extreme learning machine, it has shortcomings in training stability and can easily fall into local optimal solutions. Therefore, this study used the BES optimization algorithm to optimize the initial weights of the implicit layer of the limit learning machine and found the optimal implicit layer weights by selecting the RMSE of the ELM model constructed by the weights to be optimized for the NNI estimation results as the fitness function. This reduced the problems of the low training accuracy and poor stability of the model caused by the random generation of the weights, thus improving the convergence speed and estimation ability of the model.

By imitating the hunting strategy or intelligent social behavior of vultures when searching for fish, the BES algorithm simplifies the process of searching for the optimal solution into three stages: selecting an area, searching for prey, and diving and hunting. In the first stage, eagles choose the space with the most significant game. In the second stage, the eagle moves to the selected area in search of prey. In the third stage, the eagle swings from the best position located in the second stage and determines the best hunting spot.

The main flow of the Condor optimization algorithm is as follows: initialize population P, randomly generate the position information of each solution, and set the initial iteration number t to 0. After that, the following steps are performed for each key in the population P: evaluating new regions using formula (7), and searching and selecting parts using spiral movement. The new dive position is then assessed using Formula (8). Finally, the new work is used to dive into the prey, and formula (9) is used to evaluate the new solution. The iteration counter k is increased by two, and the previous step is repeated until the final solution in P is the best solution for solving the problem when the maximum number of iterations is reached.


(7) $$P_{new,i}=P_{best}+\alpha \times r\times \left(P_{mean}-P_{i}\right)$$

(8) $$P_{new,i}=P_{i}+y\left(i\right)\times \left(P_{i}-P_{i+1}\right)+x\left(i\right)\times \left(P_{i}-P_{mean}\right)$$

(9) $$P_{new,i}=\text{rand}\times P_{best}+x1\left(i\right)\times \left(P_{i}-C1\times P_{mean}\right)+\text{y}1\left(\text{i}\right)\times \left(P_{i}-\text{C}2\times P_{best}\right)$$

**Figure 2:**
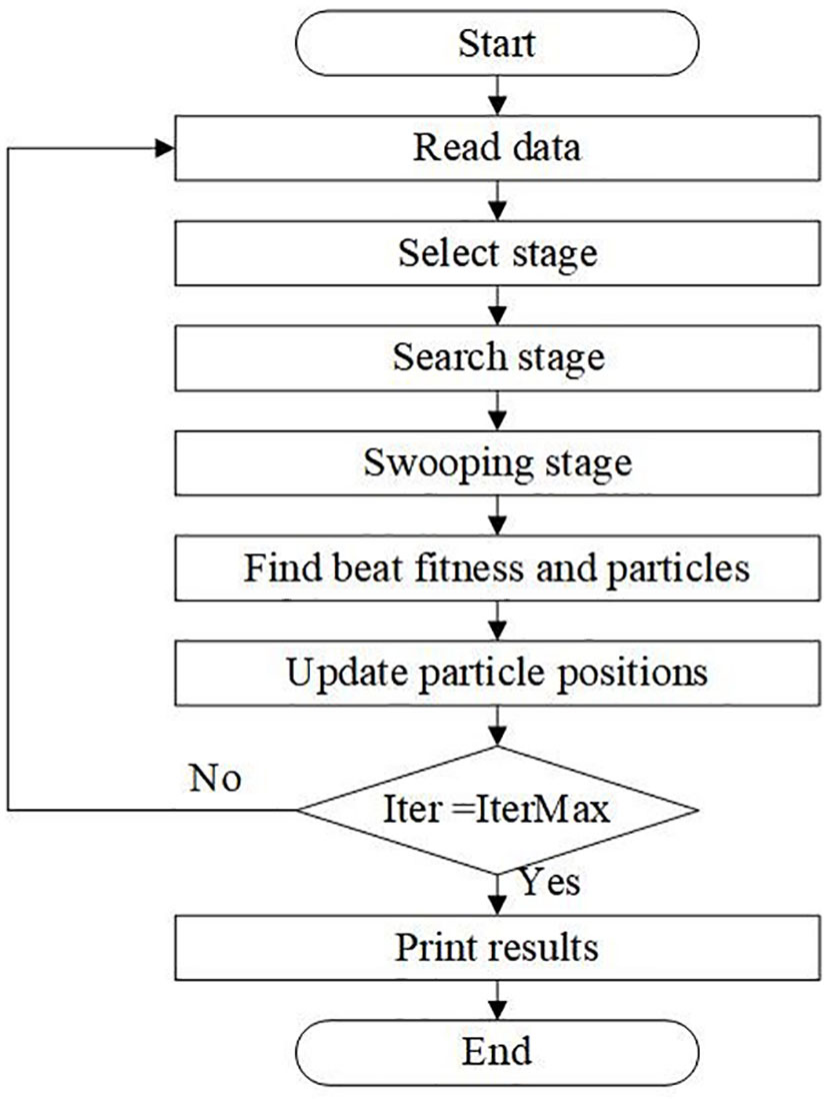
Flow Chart of BES Algorithm.

In the main work, the parameters in the above equations of the BES algorithm are defined and explained. The complete flow chart of BES algorithm optimization is shown in **Figure 2**.

#### 2.3.4 Model results evaluation

To determine the estimation ability of the model for NNI, this study evaluated the estimation accuracy and robustness of the model by the coefficient of determination R^2^ and the root mean square error (RMSE) of the model’s prediction results for the training and test sets. The calculation procedure was as follows:

, $$R^{2}=\frac{\sum_{i=1}^{n}{\left(\hat{y_{i}}-\bar{y}\right)}^{2}}{\sum_{i=1}^{n}{\left(\hat{y_{i}}-y_{i}\right)}^{2}}$$

, $$RMSE=\sqrt{\frac{1}{n}\sum_{i=1}^{n}{\left(\hat{y_{i}}-y_{i}\right)}^{2}}$$

where $\hat{y_{i}},\;\bar{y},\;\text{and}\;y_{i}$ are the measured, mean, and estimated values of rice NNI, respectively, and n is the number of samples.

## 3 Result and analysis

### 3.1 Calculation of plant N nutrient index

#### 3.1.1 Statistical analysis of plant nitrogen concentration and AGB

The basic information on plant nitrogen concentration and AGB during the whole growth period of rice is shown in **Table 1**.

**Table 1:** Plant N concentration and AGB statistics.

Indices	Maximum values	"center"Minimum values	"center"Mean	Standard deviation
Plant nitrogen Concentration of Experiment 1 /%	5.18	1.00	2.55	1.05
Above-ground biomass of Experiment 1 /(×t·hm-2)	2.35	0.10	0.73	0.55
Plant nitrogen Concentration of Experiment 2 /%	"center"5.86	1.60	3.82	0.96
Above-ground biomass of Experiment 2 /(×t·hm-2)	1.38	0.04	0.29	0.27

**Figure 3:**
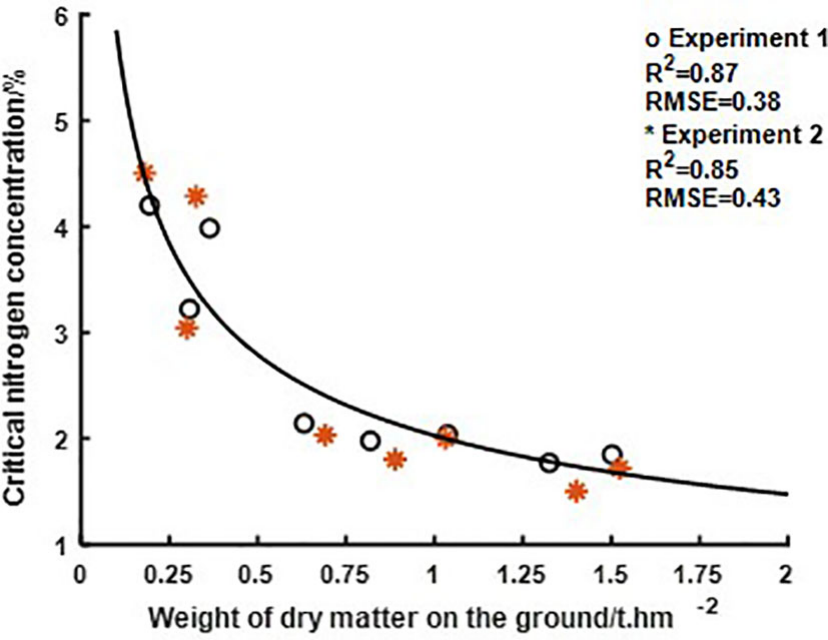
Fitting results of the critical nitrogen concentration curve. Eight points in the figure indicate the critical nitrogen concentration value corresponding to each sampling day in Experiment 1, and eight asterisks indicate the critical nitrogen concentration value corresponding to each sampling day in Experiment 2.

According to the construction method of the critical N concentration curve proposed in 2.3.1, this study fitted the regression of N concentration and AGB obtained from eight sampling days in Experiments 1 and 2 to calculate the critical N concentration value of rice for each sampling day. The critical N concentration curve according to each critical N concentration value of Experiment 1 and the corresponding AGB was then constructed, (as shown in **Figure 3**), in which the AGB and N concentration of Experiment Area 1 (as shown in **Table 2**) and the critical N concentration values of Experiment 2 were applied as validation. The calculated curve equations were as follows.

**Table 2:** AGB and N values for each period and each gradient of Experiment 1.

Indices	AGB of N0 /(×t·hm^-2^)	N of N0 /%	AGB of N1 /(×t·hm^-2^)	N of N1 /%	AGB of N2 /(×t·hm^-2^)	N of N2 /%	AGB of N3 /(×t·hm^-2^)	N of N3 /%	AGB of N4 /(×t·hm^-2^)	N of N4 /%
Day1	1.730	3.841	1.807	3.983	1.891	4.106	1.932	4.560	1.954	4.749
Day2	2.361	2.400	2.652	2.744	2.872	2.922	3.040	3.342	3.100	3.501
Day3	3.418	2.082	2.785	2.872	3.210	3.274	3.586	3.325	3.691	3.572
Day4	3.185	1.836	5.151	1.998	6.133	2.174	6.258	2.302	6.363	2.431
Day5	6.052	1.555	7.369	1.803	7.720	1.897	8.083	2.056	8.287	2.234
Day6	6.752	1.603	7.669	1.781	8.965	1.885	10.159	1.986	10.583	2.104
Day7	7.694	1.177	9.350	1.450	10.942	1.507	12.964	1.751	13.526	1.854
Day8	7.152	1.283	10.962	1.536	12.364	1.667	14.695	1.845	15.345	1.970


$${\text{N}}_{\text{c}}=2.03{\text{DM}}^{-0.46}$$

In Experiment 1, the determination coefficient R^2^ was 0.87, and the RMSE was 0.38. In Experiment 2, the R^2^ was 0.85, and the RMSE was 0.43, which were close to each other. Curves a and b were 2.03 and −0.46, respectively.

### 3.2 Spectral data processing

**Figure 4:**
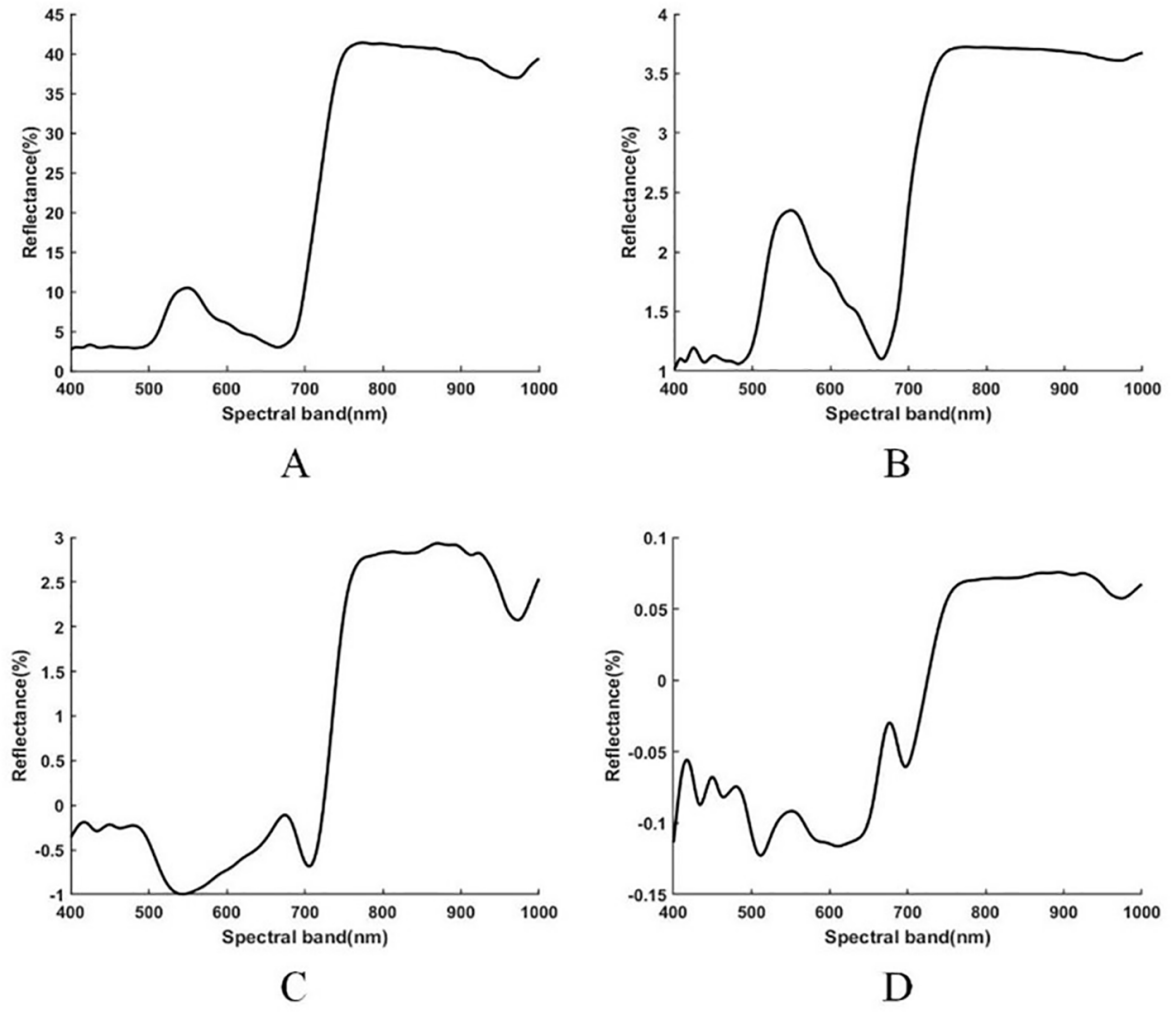
Spectral reflectance of raw, logarithmic, difference, and log-difference spectra [**(A–D)** respectively].

In this study, first, according to the NNI, the sample spectrum with an NNI equal to approximately 1 on each sampling day was selected as the standard spectrum. According to the method proposed in 2.3. 2, the difference transformation between the original spectrum and logarithmic spectrum of rice was carried out. The transformed spectrum is shown in **Figure 4**. The results showed that the original spectrum and logarithmic spectrum had a similar change trend, but the slope was higher near the blue band of 480–550 nm, and the absorption peak of the blue band was more obvious. After the difference transformation between the two spectra, the absorption peak of the red edge disappeared, and the spectrum of 400–700 nm changed into multiple segments, which is different from the original spectrum, and the spectrum trend of 700–1000 nm was unchanged.

According to the method proposed in 2.3. 3, the original spectrum, logarithmic spectrum, difference spectrum, and logarithmic difference spectrum were taken as input variables, and the spectral features were extracted by AE. The linear relationship between the feature extraction results of the AE and NNI was analyzed by MLR, and the model parameters with the best fitting degree were selected. According to the extracted results of R^2^ and RMSE in the MLR model, the final original spectrum, logarithmic spectrum, and the difference spectrum, the desired average activation of the hidden units was 0.1, the weight decay parameter was 0.001, the weight of the diversity penalty term was 3, and the number of features was 45, 37, 40, and 30, respectively. The estimated results of MLR are shown in **Table 3**.

**Table 3:** NNI estimation from encoder feature extraction results to MLR.

Transformation spectrum	R^2^of Training set	RMSE of Training set	R^2^of Test set	RMSE of Test set
Original spectrum	0.490	0.110	0.394	0.124
Logarithmic Spectrum	0.471	0.116	0.477	0.095
Difference Spectrum	0.532	0.131	0.428	0.121
Log difference spectra	0.612	0.116	0.620	0.104

### 3.3 Nitrogen nutrient index estimation model construction

In this study, the original spectra obtained in 3.2 and the feature extraction results of the difference-transformed spectra were used as input variables to construct ELM and BES-ELM-based models for estimating the NNI of rice, respectively. The influence of the difference transformation on the spectral estimation results was assessed according to the model estimation results.

#### 3.3.1 Estimation model of the rice NNI based on raw spectra

**Figure 5:**
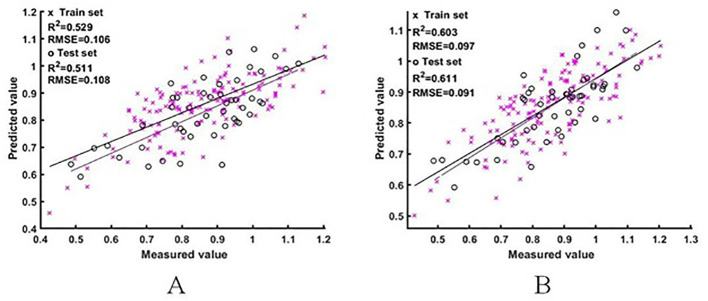
NNI estimation results based on the original spectra. (**A** is the ELM model estimation result, **B** is the BES-ELM model estimation result).

The two sets of raw spectral features were used as input variables to construct the ELM and BES-ELM based NNI estimation models, respectively, with the mapping function of Sigmoid and the fitness function of the BES-ELM model as the validation set RMSE. The training results are shown in **Figure 5**, **6**. The BES-ELM model based on logarithmic spectra had the best estimation results, with R^2^ values of 0.695 and 0.660, and RMSE values of 0.088 and 0.077, respectively, followed by the original spectrum-based BES-ELM model with R^2^ values of 0.603 and 0.611 and RMSEs of 0.097 and 0.091, respectively, and the log-spectrum-based ELM model with R^2^ values of 0.557 and 0.520 and RMSEs of 0.106 and 0.092. The ELM model based on the original spectrum had the worst estimation results with R^2^ values of 0.529 and 0.511, and RMSE values of 0.106 and 0.108, respectively. It can be seen that the estimation effect of the BES-ELM model was significantly better than that of the ELM model, and the model optimization effect was good. The estimation effect of NNI was also improved after the spectra were logarithmically transformed.

**Figure 6:**
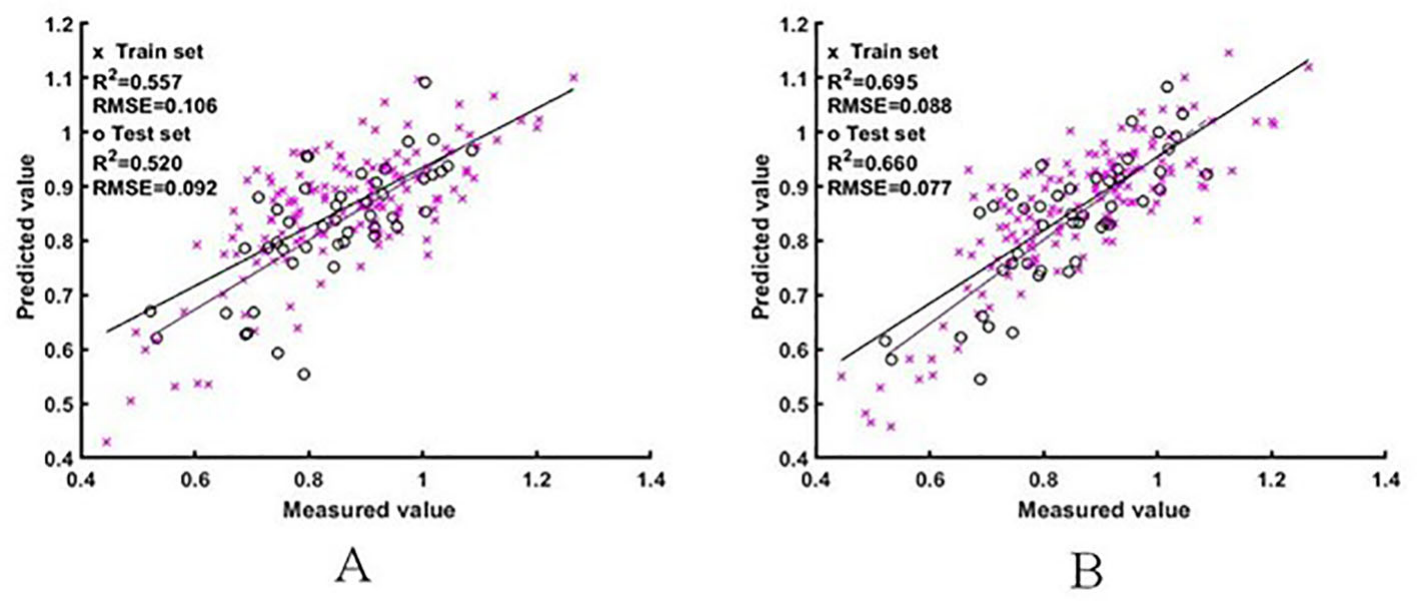
NNI estimation results based on logarithmic spectra [**(A)** is the ELM model estimation result, **(B)** is the BES-ELM model estimation result].

#### 3.3.2 A model for estimating the NNI based on the difference spectra of rice

**Figure 7:**
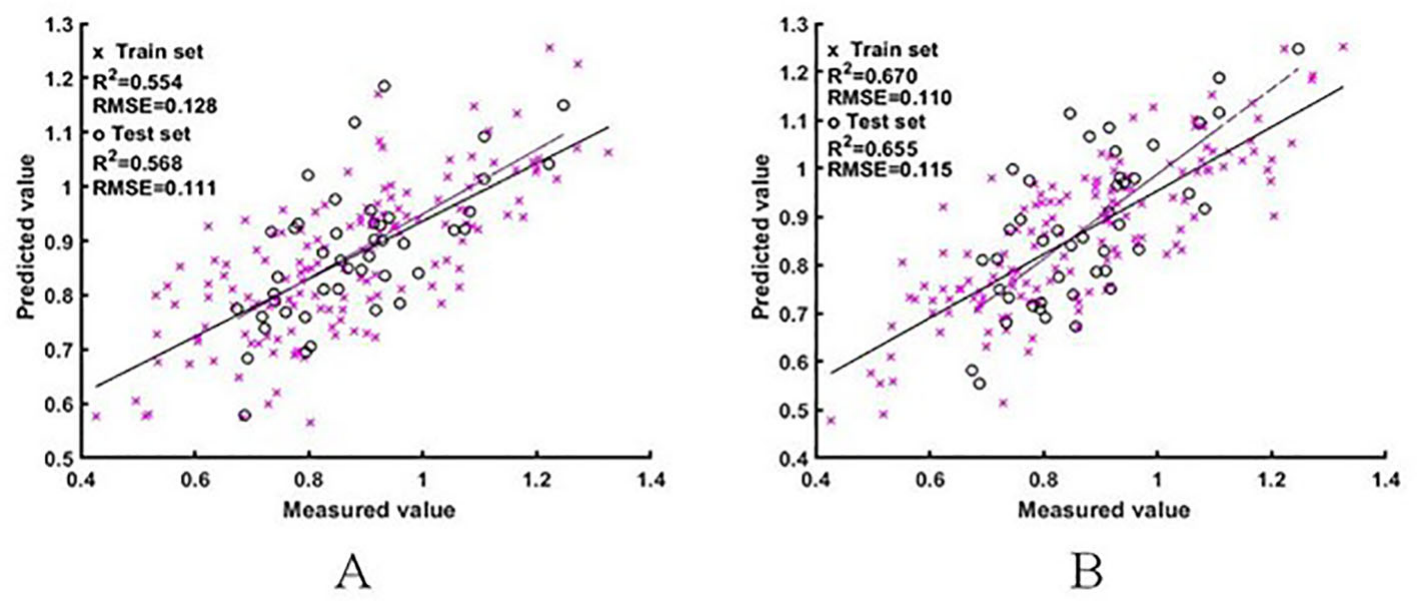
NNI estimation results based on difference spectra [**A** for ELM model estimation result, **(B)** for BES-ELM model estimation result].

Two sets of difference spectral features were used as input variables to construct the ELM and BES-ELM based NNI estimation models. The results are shown in **Figure 7**, **8**. The BES-ELM model based on log difference spectra had the highest accuracy, with R^2^ values of 0.839 and 0.837 and RMSE values of 0.075 and 0.073 for the training and validation sets, respectively. This was followed by the BES-ELM model based on difference spectra, with R^2^ values of 0.670 and 0.655 and RMSEs of 0.110 and 0.115, respectively, and then the ELM model based on the logarithmic difference spectrum, with R^2^ values of 0.628 and 0.666 and RMSE values of 0.114 and 0.099 for the training and test sets, respectively. The ELM model based on the difference spectrum had the worst estimation effect, with R^2^ values of 0.554 and 0.568 and RMSE values of 0.128 and 0.111, respectively.

**Figure 8:**
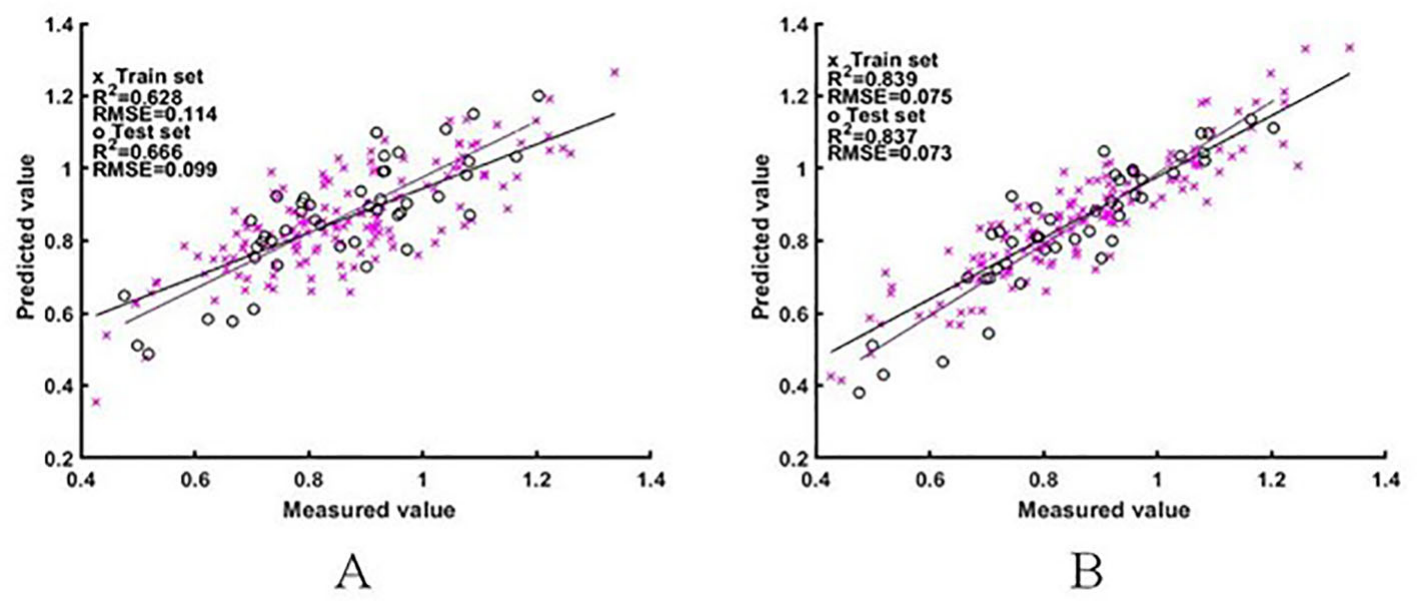
NNI estimation results based on log difference spectra [**(A)** is the ELM model estimation result, **(B)** is the BES-ELM model estimation result].

After the comparison of the model results, it can be seen that the estimation accuracy of the BES-ELM model based on log difference spectra was significantly better than the rest of the models, and in terms of the estimation models, the accuracy of the BES-ELM-based estimation models was better than that of the ELM-based models. In terms of the input spectra, the estimation results of both the original spectra and the log spectra were improved after the difference transformation, and the improvement of the log spectra was significantly better than that of the original spectra. The estimation results of the logarithmic difference model were also better than those of the remaining spectra.

### 3.4 Analysis of model estimation ability in different NNI intervals

**Figure 9:**
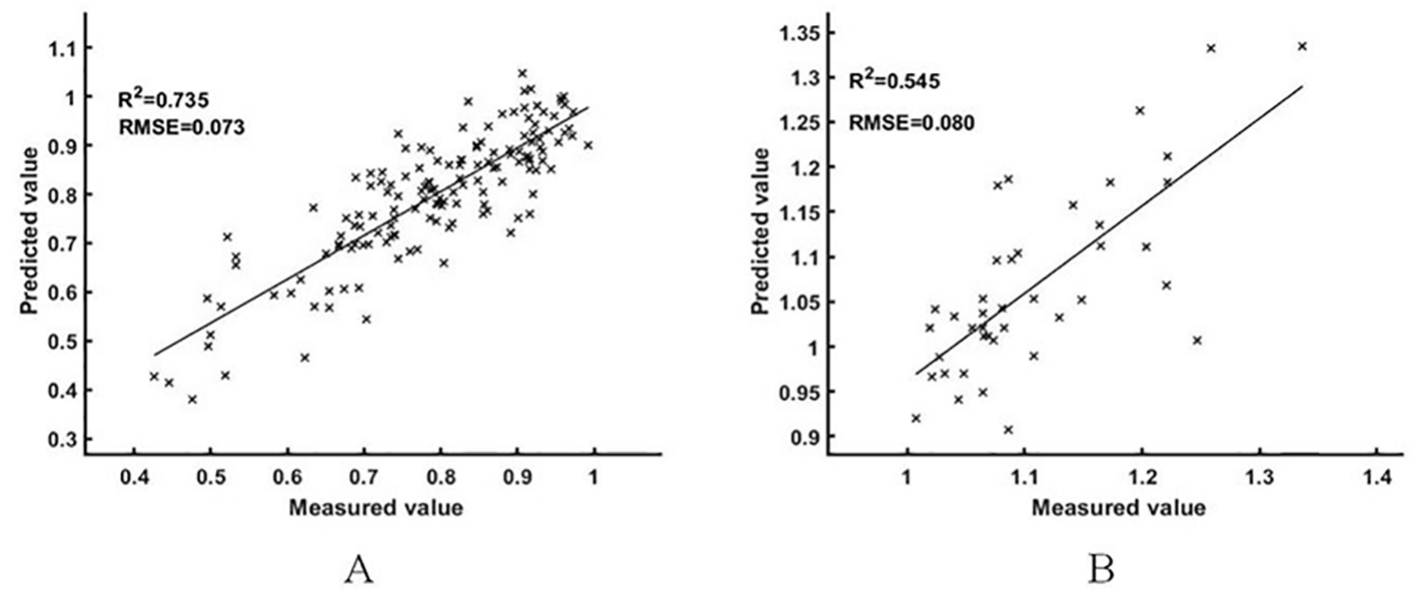
Different N nutrient index intervals [**(A)** for the N-deficient group, **(B)** for the N-rich group].

To explore the ability of the spectral transformation method to estimate the NNI of rice under different nitrogen nutrition statuses, the data were divided into a nitrogen deficit group (NNI< 1) and nitrogen-rich group (NNI ≥ 1) according to the NNI of the training set and test set, and the estimation results of the rice NNI model based on logarithmic difference spectrum and BES-ELM model in two groups were analyzed, respectively. The results are shown in **Figure 9**, in which the sample sizes of the nitrogen deficit group and nitrogen-rich group were 157 and 51, respectively. The R^2^ and RMSE values of the overall estimation results were 0.851 and 0.074, respectively; the R^2^ and RMSE values of the nitrogen deficit group were 0.735 and 0.073, respectively; and the R^2^ and RMSE of the nitrogen-rich group were 0.545 and 0.080, respectively. Comparative analysis showed that the ability to estimate the NNI of rice in the nitrogen deficit group was better than that in the nitrogen-rich group.

## 4 Discussion

### 4.1 Mechanism of estimating the NNI

To construct the diagnostic criteria of rice nitrogen nutrition at the field scale, this study selected the critical nitrogen concentration curve crop rice nitrogen nutrition status evaluation criteria according to the studies of Yuan et al. (2020) and Li et al. (2022). Since the critical nitrogen concentration curve was proposed (Justes et al., 1994), and following an in-depth study by relevant scholars (Lemaire et al., 2008), it has been proven to be an adequate diagnostic standard for nitrogen nutrition for many crops, including rice (Tremblay et al., 2011; Wang et al., 2020). Presently, field sampling and spectral inversion are mainly used to obtain NNI information on rice. Although field sampling has high diagnostic accuracy, it is challenging to meet the needs of precision agriculture in terms of acquisition cost and acquisition range. Rice hyperspectral data are low in cost and can be acquired at large scale. However, the NNI mainly describes the ratio between the actual nitrogen concentration and critical nitrogen concentration of rice and is used to evaluate the nitrogen absorption capacity of rice based on the nitrogen dilution effect and canopy growth status of rice. Additionally, this index has no physical meaning on its own, and its effect on canopy structure and canopy spectral reflectance is complex; therefore, it is more challenging to estimate NNI using only hyperspectral data than using the traditional method. The aboveground nitrogen nutrition status and canopy leaf structure of rice reach the best state under the critical nitrogen concentration, and thus these are direct factors that affect the spectral reflectance of the rice canopy. At the same time, compared with crops under non-critical nitrogen concentration, crops under critical nitrogen concentration exhibit specific differences in spectral level and nitrogen nutrition status level. Therefore, by comprehensively analyzing the construction principle of the NNI and the standard processing methods of hyperspectral data, we first transformed the hyperspectral data logarithmically to reduce the influence caused by the dimensional difference of spectral reflectance in different bands. Concurrently, to highlight the rice spectral changes based on the critical nitrogen concentration, the rice spectra under the critical nitrogen concentration in the same period (that is, rice spectra with NNI ≈ 1) were taken as the standard spectra, and the difference between the typical spectra and other spectra was amplified to improve the correlation between the transformed spectra and the NNI. The original spectrum, logarithmic spectrum, and corresponding difference transform spectrum were paired. After logarithmic transformation, the dimensional gap between various spectral bands was narrowed. Compared with the original spectrum, the value of the logarithmic spectrum in the 700–1000-nm band was no longer prominent after difference transformation, which is because logarithmic transformation narrows the dimensional gap between 400–700-nm and 700–1000-nm spectra. The slope of the curve around 700–780 nm is particularly reduced, which may be an essential reason for improving the accuracy of the estimation model.

In the feature extraction method, this study used a self-encoder to extract the features of the transformed spectrum. The self-encoder can learn the spectral data and control the output dimension freely, which is a standard algorithm for feature extraction at present. At the same time, the self-encoder, as an unsupervised feature extraction method, can extract too many redundant features, which may affect the estimation of the NNI. In this study, MLR was used to analyze the linear relationship between the feature extraction results of the self-encoder and NNI. The extraction results with the best fitting degree were selected as the model input, which avoids the problem of unclear unsupervised learning objectives to a certain extent.

### 4.2 Analysis of the modeling results

To improve the accuracy of the NNI estimation, two machine learning algorithms, ELM and BES-ELM, were selected to deal with four input spectra, and the combination with the highest estimation accuracy was chosen to construct the NNI estimation model. It can be seen from the results that in terms of spectral transformation, the estimation accuracy of the model based on the logarithmic difference spectrum was better than that of the other transform spectra. However, the difference transformation and logarithmic transformation alone had a slight improvement on the accuracy of the model, which may be because a single logarithmic transformation only narrows the relative differences in spectra of various bands, while the simple difference transformation amplifies the characteristic difference between the transformed spectrum and the critical nitrogen concentration spectrum. The dimensional differences of rice spectral reflectance in different bands are not considered, which blurs the spectral difference characteristics of different bands due to different dimensions, therefore making the actual characteristics difficult to see. The logarithmic spectrum makes the spectral characteristics of different spectra more prominent by reducing the dimensional differences between different bands, thus improving the estimation accuracy of the model. In the estimation model, BES-ELM based on the estimation model accuracy was significantly better than ELM based on the estimation model. This may be because the BES algorithm has the advantages of global solid search ability and fast convergence speed compared with traditional optimization algorithms. In this study, the RMSE of the test set was used as the fitness function of the BES algorithm. By optimizing the initial parameters of the hidden layer of ELM, the convergence speed and prediction accuracy of the algorithm were improved. The inversion effect of BES-ELM was then optimized.

At the same time, considering that based on the nitrogen dilution effect, the developmental status of rice canopy in the nitrogen-rich state does not change significantly with the elevated nitrogen nutrient status, these phenomena may lead to the reduction of correlation between spectral features and NNI and enhance the difficulty of NNI estimation, which does not occur in the rice canopy spectra in the nitrogen-deficient state. To test this conjecture and explore the analysis of model predictive ability in different NNI intervals, this study divided the data into the N deficit group (NNI< 1) and the N enrichment group (NNI ≥ 1) based on the NNI of the training and test sets, and analyzed the estimation ability of the best estimation results within the 2 groups. The results showed that the estimation ability of the N deficiency group was significantly better than that of the N enrichment group, with the R^2^ of 0.735 and 0.545, respectively, which indicated that the model was able to estimate the NNI of rice better for rice with less N fertilizer application, while the model was less able to estimate the NNI of rice with sufficient or excessive N fertilizer application. Considering that the current N nutrition diagnostic method is mainly used for precise fertilization operations for rice in N deficit state rather than N rich state, this result has less impact on actual field N fertilization management. From the research point of view, a more detailed study of rice spectra in N deficit and N enriched states could be considered in the follow-up study to investigate the response principles with canopy spectra.

### 4.3 Challenges and prospects

Several aspects of the present study can be further optimized. The first is the selection of the standard spectra. Due to the complex relationship between rice growth and development and canopy spectra, even crops with the same NNI may exhibit differences in spectra, which may also cause some interference in the selection of the standard spectra. Second, this study took the sample set size and the stability of the machine learning algorithm into account, and chose to mix the fertility samples together for model construction. Although this process satisfies the sample set requirements for subsequent modeling, the differences in canopy structure between rice at different fertility stages can cause some interference in the estimation of NNI by the model. These uncertainties also directly or indirectly reduce the accuracy and stability of the model results. Future research should combine the relationship between NNI and rice canopy structure with the existing research methods to further evaluate the principle of log-difference spectroscopy to improve the estimation results of the NNI and explore the influence of different fertility periods on the log-difference spectra of rice. A more generalized spectral transformation method should be sought to enhance the practical value of model N nutrition diagnosis.

## 5 Conclusions

In this study, rice canopy spectra were obtained by unmanned aerial remote sensing technology to estimate the NNI of rice. We determined the NNI by constructing the critical nitrogen concentration curve of rice, and performed log, difference, and log-difference transformations on the canopy spectra based on the critical nitrogen concentration spectra, and then used ELM and BES-ELM algorithms for modeling after-feature extraction by a self-encoder. Comparison of the final results indicated that the estimation model based on log-difference spectra and BES-ELM algorithm worked best, with R^2^ values of 0.839 and 0.837 and RMSEs of 0.075 and 0.073 for the training and validation sets, respectively. This study provides a new feasible approach for estimating the NNI spectra of rice.

## Data availability statement

The original contributions presented in the study are included in the article/supplementary material. Further inquiries can be directed to the corresponding author.

## Author contributions

YF, BJ, and TX designed the experiment and performed the final critical review. YF, BJ, ZJ, HZ, and YJ performed experiments and data analysis. YF, BJ, and HZ wrote the manuscript. YF, BJ, and TX provided critical review. All authors contributed to the article and approved the submitted version.
